# Prospective Validation of Modified NEXUS Cervical Spine Injury Criteria in Low-risk Elderly Fall Patients

**DOI:** 10.5811/westjem.2016.3.29702

**Published:** 2016-05-05

**Authors:** John Tran, Donald Jeanmonod, Darin Agresti, Khalief Hamden, Rebecca K. Jeanmonod

**Affiliations:** * St. Luke’s University Hospital, Department of Emergency Medicine, Bethlehem, Pennsylvania; † Carilion Clinic, Roanoke, Virginia

## Abstract

**Introduction:**

The National Emergency X-radiography Utilization Study (NEXUS) criteria are used extensively in emergency departments to rule out C-spine injuries (CSI) in the general population. Although the NEXUS validation set included 2,943 elderly patients, multiple case reports and the Canadian C-Spine Rules question the validity of applying NEXUS to geriatric populations. The objective of this study was to validate a modified NEXUS criteria in a low-risk elderly fall population with two changes: a modified definition for distracting injury and the definition of normal mentation.

**Methods:**

This is a prospective, observational cohort study of geriatric fall patients who presented to a Level I trauma center and were not triaged to the trauma bay. Providers enrolled non-intoxicated patients at baseline mental status with no lateralizing neurologic deficits. They recorded midline neck tenderness, signs of trauma, and presence of other distracting injury.

**Results:**

We enrolled 800 patients. One patient fall event was excluded due to duplicate enrollment, and four were lost to follow up, leaving 795 for analysis. Average age was 83.6 (range 65–101). The numbers in parenthesis after the negative predictive value represent confidence interval. There were 11 (1.4%) cervical spine injuries. One hundred seventeen patients had midline tenderness and seven of these had CSI; 366 patients had signs of trauma to the face/neck, and 10 of these patients had CSI. Using signs of trauma to the head/neck as the only distracting injury and baseline mental status as normal alertness, the modified NEXUS criteria was 100% sensitive (CI [67.9–100]) with a negative predictive value of 100 (98.7–100).

**Conclusion:**

Our study suggests that a modified NEXUS criteria can be safely applied to low-risk elderly falls.

## INTRODUCTION

As the population ages, elder patients presenting to U.S. medical centers are becoming increasingly frequent. Older individuals are more likely to be hospitalized after sustaining a traumatic injury and now account for up to 25% of trauma admissions.^1^ According to a retrospective review of Medicare data between 2007 and 2011, the rate of elders presenting with cervical fractures has increased from 4.6 per 10,000 to 5.3 per 10,000, while rates of hip fractures have decreased during the same time period.^2^ Associated with these cervical injuries is significant morbidity and mortality, with 30-day mortality rates of 13% in those without spinal cord injury and 28.4% in those with spinal cord injury.^2^ One-year mortality rates are respectively 24.5% and 41.7%.^2^

The National Emergency X-Radiography Utilization Study (NEXUS) criteria is a valuable clinical decision- making tool to rule out cervical spine injuries (CSI) in the general population without radiographic imaging.^3^ In the elderly population, however, there has been some reluctance to accept the reliability of NEXUS criteria^4^–^7^ despite it having a demonstrated sensitivity of 99.6%^3^ overall and a sensitivity of 100% (95% CI [97.1–100]) in the elderly cohort of the validation population.^8^

One of the criticisms of the original NEXUS criteria is the subjective nature of some of the criteria used to distinguish an interpretable patient. A paper comparing resident and attending interpretation of the NEXUS criteria found poor to fair agreement when interpreting altered mental status, focal neurologic abnormality, and distracting injury, but was limited by its relatively small sample size.^9^ Other groups have included patients with Glasgow Coma Scale (GCS) of ≥13, despite the original criteria specifying “normal alertness.”^10^ Because a large proportion of elderly patients have a GCS of 14 despite having a normal level of alertness,^11^,^12^ it is important to determine if the NEXUS criteria can be applied to this cohort.

Evans et al.^13^ demonstrated the validity of the NEXUS criteria in high-risk geriatric falls while maintaining a sensitivity of 100%. A modified NEXUS criteria was used, with more strict definitions of normal alertness and distracting injury. Normal alertness was substituted with the patient’s baseline mental status, and physical exam findings of trauma to the head or face were considered the only “distracting injuries.” With a specificity of only 12.9%, the NEXUS criteria has room for improvement to reduce unnecessary imaging.^3^ There are other studies that report a higher specificity when applying the NEXUS criteria, which range from 13%–46%.^14^ Evans et al. demonstrated an increased specificity with their modified NEXUS criteria compared to the original NEXUS criteria.^13^ However, this study was a retrospective review of higher risk elderly falls that were triaged to the trauma bay and probably represent a more injured group than those presenting to the average emergency department (ED).

Our study aims to validate this modified NEXUS criteria in a prospective study of low-risk elderly fall patients who are not triaged to the trauma bay.

## MATERIALS AND METHODS

### Study Design

This is a prospective observational cohort study of elderly fall patients at a single facility. We enrolled a convenience sample of patients and subsequently reviewed their charts. Patients or their family members or chronic care facility personnel provided verbal consent at the time of enrollment to participate in telephone contact follow up. The research protocol was reviewed and approved by the institutional review board at the study facility.

### Study Setting and Population

The study site is a Level I community trauma center that hosts an emergency medicine residency with 40 total residents. The annual ED census is about 75,000. There are about 2,100 trauma alerts annually, and 130–150 of these are for geriatric fall patients. Resident and attending physicians were educated regarding the study with monthly announcements made during weekly mandatory education time. Educational posters regarding the study were hung in physician documentation areas in the ED as well as in the nurses’ stations, and email reminders were sent to all ED medical providers at least bi-monthly.

Patients were eligible for enrollment in the study if they were 65 years of age or older and presented to the ED with a complaint related to a fall. Additionally, patients were required to be at baseline neurologic status as per their family member or chronic care facility staff. Patients were excluded if they met major trauma criteria and were triaged to the trauma bay or if they were determined to have an acute change in baseline neurologic functioning as per the physician caring for the patient, including clinical intoxication. Patients were not excluded due to dementia, aphasia, or any cognitive or neurologic deficit that was determined by the physician caring for the patient to be the patient’s baseline.

### Study Protocol and Measurements

Patients eligible for this study were identified by attending and resident physicians working in the ED. When an eligible patient presented for care, the physician caring for the patient would assess whether the patient was at baseline neurologic function. Then he or she would ask for verbal consent from the patient, caretaker, or chronic care facility personnel for research associates to contact the patient, caretaker, or chronic care facility personnel by phone in follow up. The physician caring for the patient then completed a data collection form regarding presence or absence of NEXUS criteria, and signs and location of head trauma. The data collection form contained a closed list of possibilities for each question, and the provider caring for the patient was instructed to circle his or her responses. Research associated entered data into a standardized Microsoft Excel 2007 spreadsheet (Microsoft Corporation, Redmond, WA). Study patients were evaluated and dispositioned at the sole discretion of the treating physician team.

Research associates retrospectively reviewed each patient’s medical record after his or her ED visit to determine the results of any diagnostic testing, specifically radiographic imaging, the disposition decision and service, and any neurosurgical interventions during the hospitalization. Other significant traumatic injuries were also recorded. Significant traumatic injuries included visceral injuries or bony injuries. Soft tissue injuries such as abrasions, contusions, skin tears, and lacerations were not recorded.

At 4–6 weeks after the initial ED visit, a research associate called study patients or their caregivers in follow up. This was done to assure that any patients who were neither admitted and observed nor imaged were in fact uninjured by the fall. Patients who were called were queried as to how they were feeling globally as well as specifically queried as to neck pain, numbness, tingling, weakness, and the presence of other neurologic symptoms. Patients were queried about interval ED visits and their outcome. Patients with new or ongoing symptoms were encouraged to return to the ED for further evaluation. Date of follow up and patient responses were recorded.

A patient was determined to have no significant acute neck injury if the following criteria were met: 1) He/she had a negative neck computed tomography (CT) or magnetic resonance imaging (MRI) performed; 2) the patient was admitted to the hospital and had no sequelae at discharge; 3) review of his/her medical record revealed repeat hospital visits unrelated to falls with no sequelae or complaints related to the index visit; or 4) the patient had no complaints at 30 days post-injury in telephone follow up.

### Data Analysis

We analyzed data using descriptive statistics and chi square. Data were analyzed using MedCalc (©1993–2013, Ostend, Belgium), VassarStats: Website for Statistical Computation (vassarstats.net, author Richard Lowry, PhD, Professor of Psychology Emeritus, Vassar College, Poughkeepsie, NY, © 1998–2013), and Microsoft Excel 2007 (Microsoft Corporation, Redmond, Washington). With the expectation of an injury rate similar to the original NEXUS study (4.6%), we anticipated that our confidence intervals for our sensitivity calculation would be about 88–100%, with a negative predictive value of 99%.

## RESULTS

### Demographic data

We enrolled 800 patients with fall events over a 16-month period in 2011–2012. One patient fall event was excluded because the patient was enrolled in the study twice during a single visit by two different providers, leaving 799 for analysis. Four patients were lost to follow up. These four patients were included in analyses of demographic and mechanistic data, but were excluded for all NEXUS calculations and outcome data. The majority of falls were unwitnessed (62.3%) and occurred at home. The reported mechanism and position at the time of fall as well as patient demographics are shown in Table 1. Since most falls were unwitnessed, mechanism and position were largely reported by the patients themselves.

### NEXUS criteria: normal alertness, no intoxication, and no focal neurologic deficits

All enrolled patients were at their own baseline mental status, and none had new focal neurologic deficits as per protocol. Clinically intoxicated patients were excluded from the study. Breath alcohol and blood alcohol testing was not routinely performed in this patient cohort, and was not recorded if it was performed.

### NEXUS criteria: absence of neck tenderness

Of 678 patients for whom follow up was obtained, 85.3% had an absence of neck tenderness; 95 patients had neck tenderness (11.9%). In 22 patients (2.8%), the exam was equivocal or the patient was unable to verbalize tenderness. These patients were conservatively estimated to be NEXUS positive, and were considered to have neck tenderness for the purposes of this study.

### NEXUS criteria: absence of distracting injury

Distracting injury, defined as signs of trauma to the head or neck only, was present in 366 patients (46%). An additional 114 patients with no signs of trauma to the head or neck had orthopedic injuries (most commonly hip fracture n=31, upper extremity fracture n=27, and rib fracture n=11). These orthopedic injuries, as per protocol, were not considered distracting. Therefore, 429 geriatric fall patients (54.0%) were categorized as having no distracting injury.

### Cervical spine injuries

Four patients were lost to follow up, 329 patients (46%) underwent cervical spine computed tomography, and the remainder were either admitted and observed, called by telephone, or seen in follow up (Figure). Three patients died, one of whom had a negative CT, two of whom did not have cervical spine imaging and were excluded from further analyses. Eleven cervical injuries were found: six patients had isolated injuries to C1 or C2, one had an injury to C1 and C7, and the remainder had lower CSI (Table 2). None of the patients required operative intervention.

Seven of the injured patients had cervical spine tenderness and 10 had signs of trauma to the head, most commonly to the face or frontal area (Table 3). Using the patient’s personal baseline mental status rather than GCS and using signs of trauma to the head as the only distracting injury, NEXUS performed well in this population, with a sensitivity of 100% (67.9–100%) and a negative predictive value of 100% (98.7–100%). The specificity of NEXUS in this population was 47.7 (44.2–51.3).

## DISCUSSION

Approximately 2.5 million elderly falls are treated in the ED every year, but only about 10% of these falls result in significant injury.^15^,^16^ Although it is important to not miss injuries when present, the majority of evaluated elderly patients will have negative evaluations. The NEXUS criteria aid in determining which patients need imaging, but case reports^4^,^5^ of missed injuries in elderly patients to whom the criteria have been applied have led to a number of providers who reflexively apply the Canadian C-Spine Rule of imaging to anyone greater than age 64. When this is applied to an exclusively elderly population, the specificity of the test approximates the disease prevalence and because of the low incidence of disease in a low-risk geriatric population, application of liberal imaging criteria where everyone is imaged results in an unacceptably low specificity.

Similar to the NEXUS cohort, our study population had a low rate of CSI with only 1.4% of the subjects with abnormalities seen on imaging. In the cohort of the NEXUS criteria that included only geriatric patients, the reported incidence of 4.6% was approximately twice that of the larger all-age cohort. Our reported incidence is likely lower because we specifically looked at a low-risk population that was not triaged to the trauma bay. Evans et al. reported an injury rate of 7.8%, but their sample was from a high-risk elderly fall population that was triaged to the trauma bay.^13^ We think that the incidence of injury in our cohort accurately reflects the experience of most U.S. EDs.

Using a modified NEXUS criteria, no CSI was missed resulting in a 100% sensitivity for our low-risk elderly fall population. In addition, our study demonstrated an improved specificity of 47.7%, compared to the 12.9% of the original NEXUS study,^3^ the 14% in the elderly cohort of NEXUS^8^, and the 7.4% reported in a higher risk cohort of elder patients.^13^ Also significant was the demonstration that when the modified NEXUS criteria were applied to a low-risk elderly patient population, the specificity was significantly higher than the 14% when the modified NEXUS criteria that was applied to elders in a trauma bay.^13^ This reported improved specificity is likely the result of the modified criteria being applied to an already narrowed cohort that excluded patients with altered mental status from their baseline and patients with focal neurologic deficits.

Forty-six percent of the subjects in this cohort had physical evidence of trauma to the face or head, and only 14% of the subjects had neck tenderness. Neck tenderness was the more specific criteria seen in patients with CSI, in which 6.0% of patients with neck tenderness were found to have CSI on imaging, while only 2.7% of subjects with trauma to the head or face had CSI on imaging. Because the cohort in this study only included patients who were at their normal level of alertness and without focal neurologic deficits and did not include any patients who were deemed to be clinically intoxicated, there is no information about these remaining NEXUS criteria.

Because elderly patients are more likely to injure the higher cervical spine^8^ there is concern that these patients may be missed because of absence of midline tenderness since the cervical spine is less superficial in this area. Of the seven patients in our dataset who had an injury to C1 or C2 (64% of all fractures), only one did not have midline neck tenderness on exam. Interestingly, only one of the 11 patients with a fracture had no signs of head trauma and the majority of visible trauma identified was found to be face or frontal area (9 out of 10 patients with evidence of trauma). Perhaps the finding of trauma to the face and head can be used as a surrogate marker to identify those with the potential for significant hyperextension that causes these injuries.

The idea that all elder patients with any trauma need to have their cervical spine imaged just based on their age defies common sense. The NEXUS criteria have been criticized because of the subjective nature of some of their diagnostic criteria. Hopefully, this study adds to the body of knowledge on the evaluation of cervical spine injury in elderly patients by suggesting that we can safely apply NEXUS and can more specifically apply it to patients who are at their normal mental status (but not necessarily GCS 15) and narrow the definition of distracting injury to include only evidence of trauma to the face and head. Importantly for emergency physicians, we were able to demonstrate that application of the NEXUS criteria and these modified NEXUS criteria to elderly patients results in a negative predictive value of 100%.

## LIMITATIONS

Several limitations were identified in this study. The elderly population enrolled in this study included only injury due to falls, but did not include other mechanisms such as motor vehicle collision or assault. Therefore, the data may not be extrapolated to all-cause trauma. This population was also a low-risk patient cohort who did not meet trauma alert criteria (Appendix A), which may differ at other facilities. As well, this study was done at a single tertiary care trauma center and the geriatric population may not reflect the experience of other facilities. With our data gathering, we attempted to be as specific as possible with closed-end choices, but with any data collection, there is always the possibility of misidentification or misclassification of variables such as tenderness or the presence or location of trauma. As stated in the discussion, our reported test specificity of a modified NEXUS criteria likely overestimates the actual test performance, because the cohort excluded those with altered mental status and focal neurologic findings (who were presumptively imaged) but should not have resulted in decreased sensitivity. Finally, and perhaps most importantly, because of the relative scarcity of CSI in this cohort the 95% confidence intervals are fairly wide, and ultimately this study deserves to be repeated prospectively across multiple institutions, including significantly more patients.

## CONCLUSION

Our study validates the use of a modified NEXUS criteria in a low-risk elderly fall population. Using “variation from baseline mental status” and “evidence of injury to the face or head” as a substitute of distracting injury resulted in a sensitivity and negative predictive value of 100%.

## Supplementary Information



## Figures and Tables

**Figure:**
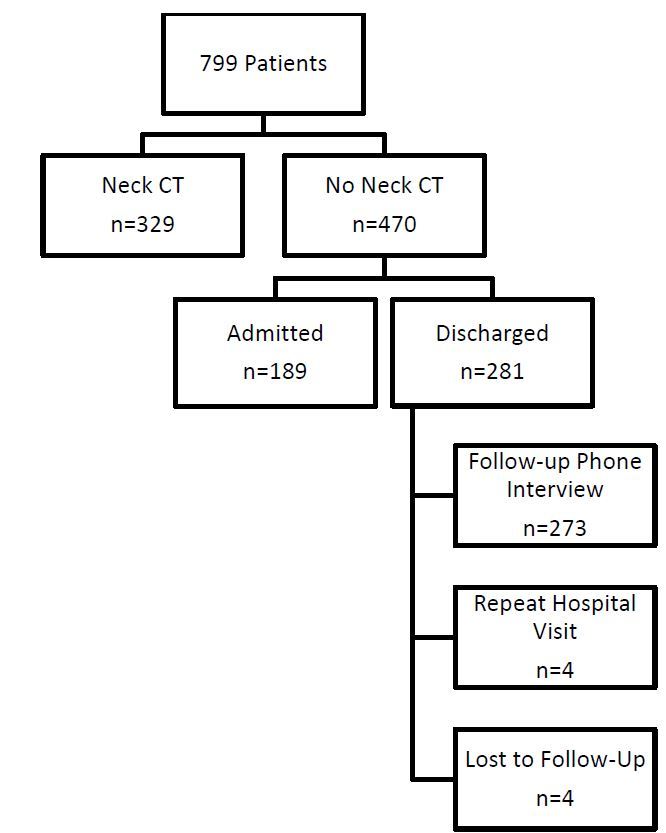
Patient imaging and follow up. *CT*, computed tomography

**Table 1:** Baseline characteristics of geriatric patients presenting to a Level I trauma center after a fall.

Characteristics	Total (n=799)
Median age (IQR)	85 (79–90)
Gender (%)	
Male	265 (33.2)
Female	534 (66.8)
Living environment (%)	
Home	450 (56.3)
Assisted living and nursing home	327 (40.9)
Other	20 (2.5)
Mechanism of fall (%)	
Trip	249 (31.2)
Loss of balance	219 (27.4)
Weakness	39 (4.9)
Dizziness	30 (3.8)
Syncope	40 (5.0)
Unsure	222 (27.8)
Position prior to fall (%)	
Standing	536 (67.1)
Seated	116 (14.5)
Lying	49 (6.1)
Climbing stairs	10 (1.3)
Unknown	88 (11.0)

**Table 2:** Cervical spine injuries in elderly fall patients.

Patient	Injury
1	Dens fracture
2	Dens fracture
3	Dens fracture
4	Dens fracture
5	C1 fracture
6	C1 fracture, occipital condyle fracture
7	C1 fracture, C7 burst
8	C4 fracture
9	C4 fracture
10	C5 fracture
11	C7 fracture

**Table 3:** Head trauma location and cervical spine injury.

Location	N=795	C spine injuries
No signs head trauma	429(54.0)	1
Head trauma location		
Face/frontal	244(30.7)	9
Parietal/occipital	52(6.5)	1
Occipital	65(8.2)	0
Unknown	5(0.6)	0

## References

[1] Haas B, Gomez D, Xiong W (2011). External benchmarking of trauma center performance: have we forgotten our elders?. Ann Surg.

[2] Cooper Z, Mitchell SL, Lipsitz S (2015). Mortality and readmission after cervical fracture from a fall in older adults: Comparison with hip fracture using national Medicare data. J Am Geriatr Soc.

[3] Hoffman JR, Mower WR, Wolfson AB (2000). Validity of a set of clinical criteria to rule out injury to the cervical spine in patients with blunt trauma. N Engl J Med.

[4] Barry TB, McNamara RM (2005). Clinical decision rules and cervical spine injury in an elderly patient: a word of caution. J Emerg Med.

[5] Collins NC, McKenzie JV (2013). The NEXUS criteria: do they withstand the test of time?. Eur J Emerg Med.

[6] Morrison J, Jeanmonod R (2014). Imaging in the NEXUS-negative patient: when we break the rule. Am J Emerg Med.

[7] Goode T, Young A, Wilson SP (2014). Evaluation of cervical spine fracture in the elderly: can we trust our physical examination?. Am Surg.

[8] Touger M, Gennis P, Nathanson N (2002). Validity of a decision rule to reduce cervical spine radiography in elderly patients with blunt trauma. Ann Emerg Med.

[9] Matteucci MJ, Moszyk D, Migliore SA (2015). Agreement between resident and faculty emergency physicians in the application of NEXUS criteria for suspected cervical spine injuries. J Emerg Med.

[10] Konstantinidis A, Plurad D, Barmparas G (2011). The presence of nonthoracic distracting injuries does not affect the initial clinical examination of the cervical spine in evaluable blunt trauma patients: a prospective observational study. J Trauma.

[11] Hustey FM, Meldon SW (2002). The prevalence and documentation of impaired mental status in elderly emergency department patients. Ann Emerg Med.

[12] Hustey FM, Meldon SW, Smith D (2003). The effect of mental status screening on the care of elderly emergency department patients. Ann Emerg Med.

[13] Evans D, Vera L, Jeanmonod D (2015). Application of National Emergency X-Ray Utilizations Study low-risk c-spine criteria in high-risk geriatric falls. Am J Emerg Med.

[14] Michaleff ZA, Maher CB, Verhagen AP (2012). Accuracy of the Canadian C-spine Rule and NEXUS to screen for clinically important cervical spine injury in patients following blunt trauma: A systematic review. CMAJ.

[15] Centers for Disease Control and Prevention, National Center for Injury Prevention and Control Web–based Injury Statistics Query and Reporting System (WISQARS).

[16] Tinetti ME, Williams CS (1997). Falls, injuries due to falls, and the risk of admission to a nursing home. N Engl J Med.

