# Supplementary material for: Prospective Validation of Modified NEXUS Cervical Spine Injury Criteria in Low-risk Elderly Fall Patients
Source: West J Emerg Med. 2016 May 5;17(3):252–7. doi: 10.5811/westjem.2016.3.29702 (PMC4899054; doi:10.5811/westjem.2016.3.29702)
Supplement: Supplementary file 1 [file wjem-17-252-s001.pdf]

Appendix 1: Adult trauma alert criteria

Vital Signs	Anatomy of Injury	Mechanism	Head Trauma in Anticoagulation	Physician/EMS Judgment
GCS <14 or combative	Flail chest	Fall > 20 feet	Abnormal neurologic exam	
SBP < 90 mmHg	Suspected pneumothorax		Loss of consciousness	
RR < 10 or > 29	2 or more long bone fractures		Amnesia	
Intubated	Amputation proximal to wrist/ankle		Headache	
Physiologic deterioration en-route	Mangled extremity		Nausea/vomiting	
	Pelvis fracture			
	Open/depressed skull fx			
	Paralysis			
